# Exploring modes of microbial interactions with implications for methane cycling

**DOI:** 10.1093/femsec/fiae112

**Published:** 2024-08-09

**Authors:** Kristof Brenzinger, Timo Glatter, Anna Hakobyan, Marion Meima-Franke, Hans Zweers, Werner Liesack, Paul L E Bodelier

**Affiliations:** Department of Microbial Ecology, Netherlands Institute of Ecology (NIOO-KNAW), Droevendaalsesteeg 10, 6708 PB Wageningen, The Netherlands; Department of Animal Ecology and Tropical Biology, Biocenter, University of Würzburg, Am Hubland, 97074 Würzburg, Germany; Core Facility for Mass Spectrometry and Proteomics, Max Planck Institute for Terrestrial Microbiology, Karl-von-Frisch-Str. 10, 35043 Marburg, Germany; Research group of Methanotrophic Bacteria, and Environmental Genomics/Transcriptomics, Max Planck Institute for Terrestrial Microbiology, Karl-von-Frisch-Str. 10, 35043 Marburg, Germany; Institute of Crop Science and Resource Conservation (INRES) , Molecular Biology of the Rhizosphere, Nussallee 13, 53115 Bonn, Germany; Department of Microbial Ecology, Netherlands Institute of Ecology (NIOO-KNAW), Droevendaalsesteeg 10, 6708 PB Wageningen, The Netherlands; Department of Microbial Ecology, Netherlands Institute of Ecology (NIOO-KNAW), Droevendaalsesteeg 10, 6708 PB Wageningen, The Netherlands; Research group of Methanotrophic Bacteria, and Environmental Genomics/Transcriptomics, Max Planck Institute for Terrestrial Microbiology, Karl-von-Frisch-Str. 10, 35043 Marburg, Germany; Department of Microbial Ecology, Netherlands Institute of Ecology (NIOO-KNAW), Droevendaalsesteeg 10, 6708 PB Wageningen, The Netherlands

**Keywords:** bacterial interaction, heterotrophs, methane oxidation, methanotrophs, protein composition, volatile organic compounds

## Abstract

Methanotrophs are the sole biological sink of methane. Volatile organic compounds (VOCs) produced by heterotrophic bacteria have been demonstrated to be a potential modulating factor of methane consumption. Here, we identify and disentangle the impact of the volatolome of heterotrophic bacteria on the methanotroph activity and proteome, using *Methylomonas* as model organism. Our study unambiguously shows how methanotrophy can be influenced by other organisms without direct physical contact. This influence is mediated by VOCs (e.g. dimethyl-polysulphides) or/and CO_2_ emitted during respiration, which can inhibit growth and methane uptake of the methanotroph, while other VOCs had a stimulating effect on methanotroph activity. Depending on whether the methanotroph was exposed to the volatolome of the heterotroph or to CO_2_, proteomics revealed differential protein expression patterns with the soluble methane monooxygenase being the most affected enzyme. The interaction between methanotrophs and heterotrophs can have strong positive or negative effects on methane consumption, depending on the species interacting with the methanotroph. We identified potential VOCs involved in the inhibition while positive effects may be triggered by CO_2_ released by heterotrophic respiration. Our experimental proof of methanotroph–heterotroph interactions clearly calls for detailed research into strategies on how to mitigate methane emissions.

## Teaser

The interaction between methanotrophs and heterotrophs can have strong positive or negative effects on methane consumption and growth, depending on the species interacting with the methanotroph.

## Introduction

Methane (CH_4_) is Earth’s second-most important greenhouse gas (GHG) after CO_2._ The current atmospheric CH_4_ concentration of 1.896 parts per million (ppm*_v_*) is the highest in at least 80 000 years (Masson-Delmotte et al. 2021) and in recent years, the increase has even accelerated (Fletcher and Schaefer 2019, Masson-Delmotte et al. 2021). Global CH_4_ emissions contribute 15%–35% to total global radiative forcing (Masson-Delmotte et al. 2021) of which the largest part originates from agricultural (i.e. rice paddies) and natural wetlands (Saunois et al. 2020). In fact, the recent increase in atmospheric CH_4_ concentrations is likely associated to higher biological CH_4_ production and emission from wetlands due to warmer and wetter climate (Peng et al. 2022). These biological feedbacks to global warming, often involving microbially driven biogeochemical reactions, are poorly understood, necessitating further investigations of the controlling factors of the main processes involved (Cavicchioli et al. 2019, Gedney et al. 2019). The only biological sink for CH_4_ globally are CH_4_-oxidizing microbes (Bodelier et al. 2019), which exert a profound influence on CH_4_ dynamics in various environments. Because of their specialized lifestyle, utilizing CH_4_ as their primary carbon and energy source, they are called methanotrophs.

Methanotrophs, encompassing diverse taxa capable of degrading CH_4_ with and without oxygen (O_2_), have garnered considerable attention due to their role in mitigating CH_4_ emissions from natural and anthropogenic sources (Bodelier et al. 2019, Guerrero-Cruz et al. 2021). These microbes employ unique enzymes, including CH_4_ monooxygenase (MMO), to oxidize CH_4_ to methanol, serving as the foundation for their metabolic activities. There are two types of MMO: the soluble CH_4_ monooxygenase (sMMO) and the particulate CH_4_ monooxygenase (pMMO). The sMMO is found in the cytoplasm of methanotrophic bacteria and is notable for its ability to oxidize a broad range of substrates besides CH_4_. It contains a nonheme iron center and operates under a wide range of environmental conditions. The pMMO is embedded in the cellular membranes of methanotrophic bacteria. It primarily oxidizes CH_4_ to methanol with high specificity and contains copper centres that are crucial for its catalytic activity. Concurrently, heterotrophic microorganisms, comprising a vast array of bacteria and fungi, derive their energy from organic carbon substrates, displaying remarkable metabolic versatility and ecological adaptability across diverse habitats.

However, recent research has unveiled a complex interplay between methanotrophs and heterotrophic microorganisms. While shedding light on their intricate interactions and their broader ecological significance, this intriguing area of research revealed a spectrum of symbiotic, competitive, and syntrophic relationships. (Stock et al. 2013, Ho et al. 2014, 2017, Padmavathy 2017). Earlier paradigms predominantly viewed these two functional guilds as operating independently, but mounting evidence suggests intricate metabolic exchanges and mutual dependencies among them (Stock et al. 2013, Ho et al. 2014, Veraart et al. 2018). For instance, heterotrophs can utilize intermediates produced during CH_4_ oxidation by methanotrophs or vice versa (e.g. cobalamin, organics, or CO_2_), thereby fostering a dynamic network of cross-feeding interactions within microbial consortia (Stock et al. 2013, Ho et al. 2014, Yu et al. 2017). Understanding the mechanisms underpinning the mutualistic or antagonistic relationships between methanotrophs and heterotrophs holds immense significance in elucidating the biogeochemical cycles of carbon and energy fluxes within ecosystems. These interactions potentially influence CH_4_ consumption rates, substrate availability, and community structure, consequently impacting ecosystem resilience and functioning (Ho et al. 2017).

A potential way of communication between the methanotrophic and heterotrophic guilds, especially in case of environmental physical separation, could be via volatile organic compounds (VOCs) (Audrain et al. 2015, Schmidt et al. 2015, Tyc et al. 2015, Veraart et al. 2018). Plants and soil microorganisms in the rhizosphere can produce and release various VOCs such as ethylene, CH_4_, and volatile terpenes (Diyapoglu et al. 2022, Srikamwang et al. 2023). Microbial VOCs are known to act as signalling molecules, facilitating communication, and modulating microbial behaviour (Korpi et al. 2009, Weisskopf et al. 2021), which can even act over cm’s distances in soil (Schulz-Bohm et al. 2018). These VOCs play important roles in plant–microbe interactions, defence mechanisms, and signalling processes (Ortíz-Castro et al. 2009, Bitas et al. 2013), but can also have ecological consequences for biogeochemical cycles (de la Porte et al. 2020).

In high CH_4_ environments like rice paddy soils, landfills, or chemoclines of lakes, with a high turnover of CH_4_, VOC-mediated interactions between methanotrophs and heterotrophs can have profound influence on CH_4_ cycling and emission to the atmosphere. Rice paddies or stratified lakes are major sources of CH_4_ due to anoxic conditions that are conducive to the production of CH_4_ (Minami and Neue 1994, Cao et al. 1996, Schubert et al. 2010, Fuchs et al. 2022). Methanotrophs can help to mitigate these emissions by consuming the CH_4_ that is produced, thus reducing the amount that escapes into the atmosphere, but can also play a key role in the biogeochemical cycling of carbon and other nutrients (Guerrero-Cruz et al. 2021, He et al. 2023). However, the extent to which interactions with other microbes modulate methanotrophic activity as well as the underlying mechanisms have sparsely been investigated. Considering the crucial environmental role of methanotrophs, it is therefore necessary to assess and understand the modes of interaction involved. In this context, we aimed to assess the interplay between methanotrophs and heterotrophs, integrating various analytical techniques, including proteomics, volatile analysis, and measurements of bacterial growth and CH_4_ oxidation. This multidisciplinary approach aims at identifying and disentangle the complex volatolomes and elucidate the impact of the volatolomes on the methanotroph activity (process measurements) and proteome. Volatile analysis was employed to investigate the production and consumption of specific VOCs during microbial interactions. Proteomics, a powerful tool in systems biology, was employed to elucidate the protein expression patterns of methanotrophs and heterotrophs when confronted with each other. By comparing the proteomes of these organisms, we identified key proteins involved in CH_4_ oxidation, carbon metabolism, and interspecies communication. This allowed for a better understanding of the molecular basis of the interactions between methanotrophs and heterotrophs and will shed light on our earlier, more descriptive findings (Veraart et al. 2018). The findings of this research will contribute to our knowledge of CH_4_ cycling in various environments, ultimately helping to develop strategies for mitigating CH_4_ emissions and understanding the ecological roles of methanotrophs and heterotrophs in microbial communities.

## Materials and methods

### Experimental design

In this study, we analysed the interaction of heterotrophs with our model methanotroph organism (*Methylomonas* spp. LL1; de Assis Costa et al. 2021), as well as the effect of elevated CO_2_ concentrations on the LL1 strain, on its activity, volatile production, and growth. In addition to *Methylomonas* spp. LL1, we used two heterotrophs (*Microbacterium oxydans* H1 and *Exiguobacterium undae* H5; details about isolation see Veraart et al. (2018) and Krause et al. (2015). The heterotrophs were isolated from methanotrophic enrichment cultures, which were inoculated with wetland sediment and hence, they co-occur naturally with methanotrophs. *Methylomonas* spp. LL1 was also isolated from a wetland soil (Bodelier et al. 2012, 2013, de Assis Costa et al. 2021) and was used on the basis of environmental origin. *Exiquobacterium* was chosen because of the positive effect on methanotrophic growth and activity while *Microbacterium* was chosen from our collection of wetland enrichment isolates because these strains have been demonstrated in pilot incubation to have an inhibitory effect on methanotrophic growth (Cordovez et al. 2018). *Methylomonas* sp. LL1 was incubated either alone or together with one of the heterotrophs. In addition, we incubated *Methylomonas* sp. LL1 under elevated CO_2_ (5%) atmosphere (Vorobev et al. 2011). An overview is provided in Table 1. We used a similar incubation approach as in a previous study (Veraart et al. 2018) in which methanotrophs were grown in nitrogen mineral salts (NMS) medium, while heterotrophs were incubated in dilute tryptic soy broth (0.1% TSB) medium. In brief, preincubations were conducted at 25°C with shaking at 145 r m^−1^ in the dark using 50 ml Erlenmeyer flasks with either NMS medium for the methanotroph (Whittenbury et al. 1970) or TSB medium for the two heterotrophs (Johnson and Kelso 1983). From these precultures two parallel experiments were conducted, one for the measurement of CH_4_ uptake rates and the other one for the measurement of volatile production, bacterial growth, and proteomics analyses of the methanotroph. In total the experiment was run for 2 weeks.

**Table 1.** Experimental setup with all used incubation combinations.

	Plates compartment 1 NMS agar	Plates compartment 2 TSB agar	Headspace
1	*Methylomonas* sp. LL1	Empty	Air + 20% CH_4_
2	*Methylomonas* sp. LL1	*M. oxydans* H1	Air + 20% CH_4_
3	*Methylomonas* sp. LL1	*E. undae* H5	Air + 20% CH_4_
4	*Methylomonas* sp. LL1	Empty	Air + 20% CH_4_/ + 5% CO_2_
5	Empty	*M. oxydans* H1	Air + 20% CH_4_
6	Empty	*E. undae* H5	Air + 20% CH_4_
7	Empty	Empty	Air + 20% CH_4_

### CH_4_ oxidation assay

CH_4_ oxidation was measured using two-compartment Petri dishes (Greiner, catalogue number 635102), one side containing 12.5 ml 0.1% TSB-agar, the other side containing 12.5 ml NMS-agar. Nutrient composition of the agar was as described in Veraart et al. (2018), but with 15 g l^−1^ agar added (Bacto agar, for NMS, Merck agar for TSB). In total we prepared 28 plates, with four replicates for each combination (Table 1). In treatments which contained heterotrophs, 50 µl of the respective liquid preculture [diluted to optical density at 600 nm (OD_600_) of 0.5] was spread on the TSB side of the plates. Following, 50 µl of the *Methylomonas* sp. LL1 preculture was spread on the NMS side of the plates (diluted to OD_600_ of 0.5). Plates were preincubated at 25°C in gas-tight jars for 5 days with a headspace containing 20% CH_4_ in air. After preincubation, individual plates were opened, and vented for 30 min in a fume hood to remove all gas from the previous incubation. Afterwards plates were sealed again and placed in closed flux chambers (V: 172 ml) (Ho et al. 2011), containing a sampling port with a silicon rubber septum. For the CH_4_ oxidation measurement alone one % of CH_4_ (10 000 ppm*_v_*) was added to the headspace of each chamber (treatment 4 (Table 1) received 5% additional CO_2_), after which CH_4_ concentrations were measured by sampling the headspace using a Vici precision sampling 250 µl syringe equipped with a side port needle (BGB Analytik, Schloßböckelheim, Germany) in a period of ∼29 h. CH_4_ measurements were conducted using an Ultra gas chromatograph (GC) (Interscience, Breda, The Netherlands) equipped with a flame ionization detector and a Rt-Q-Bond (L; 30 m, ID; 0.32 mm, Restek, Interscience) capillary column. Helium was used as a carrier gas, and oven temperature was set at 80°C. Chromeleon™ Chromatography Data System 7.1 (CDS, Thermo Fisher Scientific) Software was used to analyse the gas chromatograms obtained from the GC. CH_4_ oxidation rates were calculated using linear regression of headspace CH_4_ concentrations retrieve between 2.5 and 28.5 h, to avoid variation in measurements directly after starting incubation and subsequently capturing maximum rates.

### Volatile analysis

Volatiles were analysed for all three strains alone, the combinations of *Methylomonas* sp. LL1 with both heterotrophs and the combination of *Methylomonas* sp. LL1 with additional CO_2_. The same treatments as for the CH_4_ oxidation measurements were applied (Table 1), each with four replicates. Split plates as described above were inoculated with precultures growing in the mid-exponential phase, spread with 50 µl of each heterotroph on one side and spotted with seven droplets of 8 µl of *Methylomonas* sp. LL1 on the other side. Plates were preincubated for 5 days in gas-tight jars containing 20% CH_4_ in the headspace and additional 5% CO_2_ in the *Methylomonas* sp. LL1 + CO_2_ treatment. After preincubation, the plates were placed in volatile-trapping chambers (Ho et al. 2011, Veraart et al. 2018), closed with a butyl septum through which CH_4_ and CO_2_ could be injected, and a steel volatile trap containing 150 mg Tenax TA and 150 mg Carbopack B (Markes International, Ltd., Llantrisant, UK) was inserted (Tyc et al. 2015). Plates containing traps were incubated in the dark at 25°C for 48 h, after which volatile traps were removed, rapidly capped, and stored at 4°C until further analysis within 2 weeks. Volatile analyses were performed on a Quadrupole Time of Flight GC/MS (hereafter GC-Q-TOF, Agilent 7890B GC, Agilent 7200A/B Q-TOF) as described in Veraart et al. (2018). For the volatolomics analysis, the acquired raw mass spectrometry (MS) data was extracted to m/z format using MassHunter Qualitative Analysis Software V B.07.00 (Agilent Technologies, Santa Clara, CA, USA). The m/z data was processed with MZMine V 2.36 (Copyright © 2005–2012 MZmine Development Team) to create a m/z and peak intensity table that could be used as input file for MetaboAnalyst 4.0 software (http://www.metaboanalyst.ca/MetaboAnalyst) (Pluskal et al. 2010, Lankenau et al. 2015, Chong et al. 2018). Before the statistical analysis, the data was filtered using Interquartile range (IQR) and normalized by the log transformation with automatic scaling (Ossowicki et al. 2020). The VOCs raw data can be received upon request due to the size of the dataset (∼650 GB).

The data displayed in Figs 4 and 5 are normalized and display relative mass intensity values of compounds detected, relative to the empty plates. Compounds detected in the empty plates should also appear in the treatments. However, since the ‘contaminating/background’ compounds are in most cases (see Supplementary Table 6) in low intensities present they will be either detected or not. Whether a nonmicrobiologically produced compound is detected or not may depend on the experimental handling but also potential degradation by the microbes in the inoculated plates. Especially, when amounts are low this can lead to zeros which will not happen in the empty plates. These inconsistencies are probably due to a combination of replication problems (i.e. degree of contamination is difficult to control in our experimental handling), caused measuring around detection limits in combination with degradation by the microbes in the experiment.

### Determination of bacterial growth and proteome analyses

Immediately following the incubation for the volatile experiment, cell material from the methanotroph and the two heterotrophs were harvested by flushing the plates with 3 ml of NMS medium or TSB medium, respectively. The cells were gently scraped off using a cell-scraper, and an aliquot of the resulting cell suspension was diluted for OD_600_ measurement. Afterwards the cell suspension of each sample was centrifuged, and the pellets were resuspended and transferred to 2 ml vials. Then all suspensions were centrifuged again and washed twice with 1x PBS after which the cell pellets were frozen using liquid N_2_ and stored at −80°C till further processing for proteomics analysis. Since the growth of *Methylomonas* sp. LL1 in the combinations with *M. oxydans* H1 was inhibited by the heterotroph, this combination was excluded from the proteomics analyses.

### Extraction, solubilization, and digestion of proteins

The proteomics sample preparation was done according to a previous study (Hakobyan et al. 2018). In brief, the frozen cell pellets were resuspended and sonicated (Hielscher Ultrasound Technology) in 2% sodium deoxycholate buffer (SDC, dissolved in 100 mM ammonium bicarbonate) in the presence of 5 mM tris[2-carboxyethyl]phosphine and subsequently incubated at 95°C for 60 min. The samples were subjected to additional 20 s rounds of sonication after 15 and 30 min of incubation. Upon the SDC treatment, all the protein samples were allowed to cool followed by incubation with 10 mM iodoacetamide at 25°C for 30 min. The resulting crude lysates were used for further digestion as described below. The concentration of proteins in each sample was measured with BCA protein assay kit (Thermo Fisher Scientific) according to the manufacturer’s instructions.

For protein digestion step, 50 μg of total solubilized protein from crude lysate samples was used. For in-solution digestion, the samples were diluted to 0.5% of solubilizing (SDC) agent in 100 mM ammonium bicarbonate buffer. LysC (0.5 µg, Wako Chemicals GmbH) was added directly to the protein extract and incubated for 4 h at 30°C, followed by trypsin (1 μg, Promega) digestion overnight at 30°C. Before LC–MS analysis, traces of SDC were precipitated using 2% trifluoroacetic acid, and all protein digests were desalted using C18 microspin columns (Harvard Apparatus) according to the manufacturer’s instructions.

### LC–MS/MS analyses, peptide/protein identification, and LFQ quantification

The LC–MS/MS analysis of protein digests was performed on a Q-Exactive Plus mass spectrometer connected to an electrospray ion source (Thermo Fisher Scientific). Peptide separation was carried out using the Ultimate 3000 nanoLC-system (Thermo Fisher Scientific), equipped with an in-house packed C18 resin column (Magic C18 AQ 2.4 µm, Dr. Maisch). The protein digest (1 µg) was first loaded onto a C18 precolumn (preconcentration set-up) and then eluted in backflush mode with a gradient from 98% solvent A (0.15% formic acid) and 2% solvent B (99.85% acetonitrile, 0.15% formic acid) to 25% solvent B over 105 min, continued from 25% to 35% of solvent B up to 135 min. The flow rate was set to 300 nl/ min. The data acquisition mode was set to obtain one high-resolution MS scan at a resolution of 60 000 (m/z 200) with scanning range from 375 to 1500 m/z followed by MS/MS scans of the 10 most intense ions (Top10 DDA). The dynamic exclusion duration was set to 30 s. The ion accumulation time was set to 50 ms (both MS and MS/MS). The automatic gain control was set to 3 × 10^6^ for MS survey scans and 1 × 10^5^ for MS/MS scans. For label-free quantitative analysis MS raw files were imported into Progenesis (Nonlinear Dynamics, version 2.0) and the output data (MS/MS spectra) were exported in mgf format. MS/MS spectra were then searched using MASCOT (v.2.5, Matrix Science) against a decoy database of the predicted proteomes from *Methylomonas* sp. LL1, *Exiguobacterium* sp. RIT341, and *M. oxydans*. The protein databases were downloaded from uniprot and evaluated for protein sequence redundancy. The following search parameters were used: full tryptic specificity required (cleavage after lysine or arginine residues); two missed cleavages allowed; carbamidomethylation (C) set as a fixed modification; and oxidation (M) set as a variable modification. The mass tolerance was set to 10 ppm for precursor ions and 0.02 Da for fragment ions for high energy-collision dissociation (HCD). Results from the database search were imported back to Progenesis to map peptide identifications to MS1 features. The peak heights of all MS1 features annotated with the same peptide sequence were summed, and protein abundance was calculated per LC–MS run. Next, the data obtained from Progenesis were evaluated using SafeQuant R-package version 2.2.2 (Glatter et al. 2012). Hereby, 1% FDR of identification and quantification as well as intensity-based absolute quantification (iBAQ) values were calculated.

Significantly up- or downregulated proteins were defined as having values of the log_2_ ratios of >1.0 or <−1.0, respectively, with *q*-value < 0.05. The 75 proteins with the highest order of significance were also visualized on a heat map in which clustering was based on Euclidian distance, using Ward’s algorithm. For visualization of similarity between the different proteomics profiles we used a principal component analysis (PCA) and permutational multivariate analysis of variance (PERMANOVA) analyses was performed to compare the differences between groups based on multivariate data (Supplementary Table 7). ClusterProfiler version 3.12.0 was used to perform functional enrichment analyses based on Kyoto Encyclopedia of Genes and Genomes (KEGG) pathways. Here, the gene set enrichment analysis (GSEA) function (Subramanian et al. 2005) was applied to determine whether an *a priori* defined set of genes based on KEGG shows statistically significant, concordant differences between the different treatments.

### Statistical analysis of growth and CH_4_ oxidation

All statistical analyses were done using R version 4.2.0 (R Development Core Team 2008). The CH_4_ uptake rate and bacterial growth from the different treatments were tested for normality by Kolmogorov–Smirnov test and homogeneity of variance by Levene’s test. If necessary, normal distribution was achieved by log-transformation of the data. Mean differences were assessed using one-way analysis of variance ANOVA followed by Tukey’s *post hoc* test. All levels of significance were defined at *P* < .05.

Statistical analysis of the volatile data was performed on the resulting peak intensity table using the MetaboAnalyst platform (Chong et al. 2018). We filtered peak intensities based on IQR, then log transformed and autoscaled them (mean-centred and divided by the standard deviation of each variable) to obtain a normal distribution. We used one-way ANOVA with Tukey’s *post hoc* test, to identify significant differences in peak intensities between samples, for each observed compound. PCA was used to visualize maximum separation between groups based on their peak intensities and permutational multivariate analysis of variance (PERMANOVA) analyses was performed to compare the differences between groups based on multivariate data (Supplementary Table 7). We constructed heat maps of peak intensities of the detected compounds in the samples to visualize differences between treatments, in which clustering was based on Euclidian distance, using Ward’s algorithm.

### Data deposition

The MS proteomics data have been deposited to the ProteomeXchange Consortium via the PRIDE partner repository with the dataset identifier PXD051964, which can be accessed with Username: reviewer_pxd051964@ebi.ac.uk and Password: bkeJRW0q.

## Results

### Bacterial growth and CH_4_ oxidation

**Figure 1.**
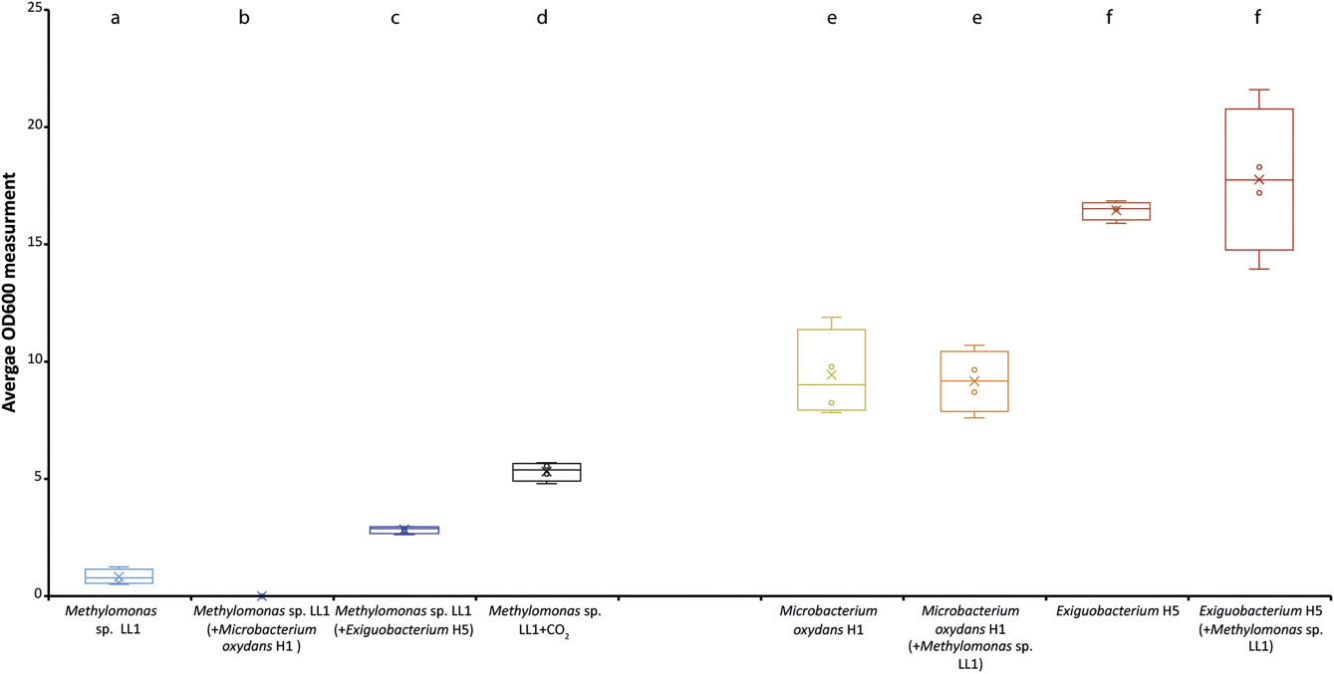
Cell growth of the six different treatments based on OD_600_ measurements. When a strain grew alone on the two-compartment agar plate, only its name is stated. If the strain grew with another strain, the name of the other strain is mentioned in parentheses below the name of the measured strain. Different letters indicate significant differences (*P* < .05) in the mean cell growth of each sample. Mean ± SD (*n* = 4). See Table 1 for further details on the six treatments.

*Methylomonas* sp. LL1 performed best on plates with the addition of CO_2_ (OD_600_ = 5.3), while no growth was observed for *Methylomonas* sp. LL1 in combination with *M. oxydans* H1 (Fig. 1; Supplementary Table 1). Even though the growth of *Methylomonas* sp. LL1 together with *Exiguobacterium* sp. H5 (OD = 2.8) was ∼47% lower compared to its growth with CO_2_, it was still 71% higher than *Methylomonas* sp. LL1 (OD = 0.8) growing alone. The two heterotrophs showed a similar growth performance, no matter whether they were incubated with or without the addition of *Methylomonas* sp. LL1.

**Figure 2.**
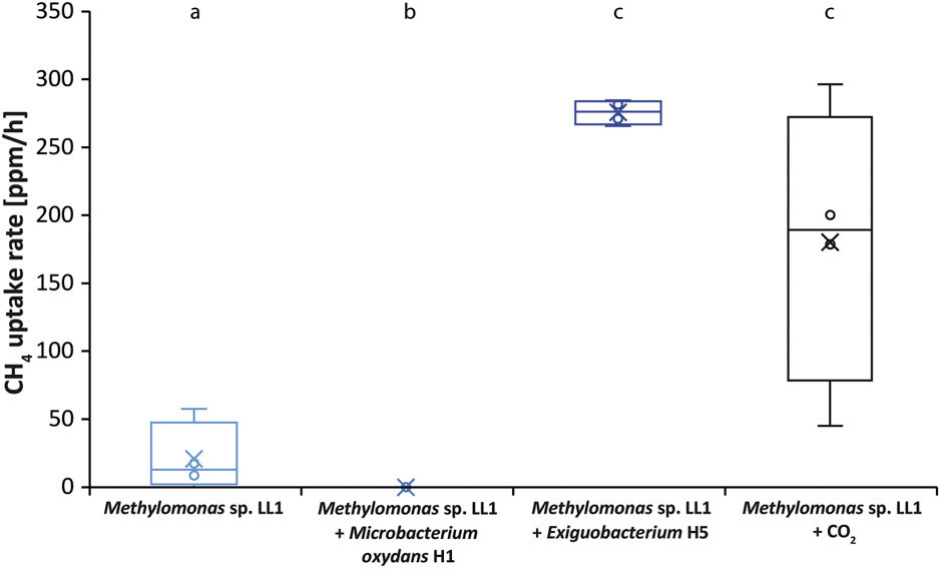
CH_4_ uptake rate for *Methylomonas* sp. LL1 incubated alone, in combination with the two heterotrophs, and with the addition of 5% CO_2_ in the headspace. Since the growth of *Methylomonas* sp. LL1 was inhibited by *M. oxydans*, no CH_4_ uptake rate was measurable. Same letters indicate similarity in CH_4_ uptake rate, different letters indicate significant differences (*P* < .05). Mean ± SD (*n* = 4.)

The highest CH_4_ uptake rate (Fig. 2; Supplementary Table 2) of *Methylomonas* sp. LL1, was observed when growing together with *Exiguobacterium* sp. H5 (mean: 275 ppm h^−1^), which was almost 30 times higher as compared to growing alone (mean: 15 ppm h^−1^). The CH_4_ consumption rate with additional CO_2_ added (mean: 180 ppm h^−1^) tended to be lower than the rate within the presence of *Exiguobacterium* sp. H5. However, due to the high variability between replicate samples this difference was not significant (*P* > .05).

### Proteomic analyses

**Figure 3.**
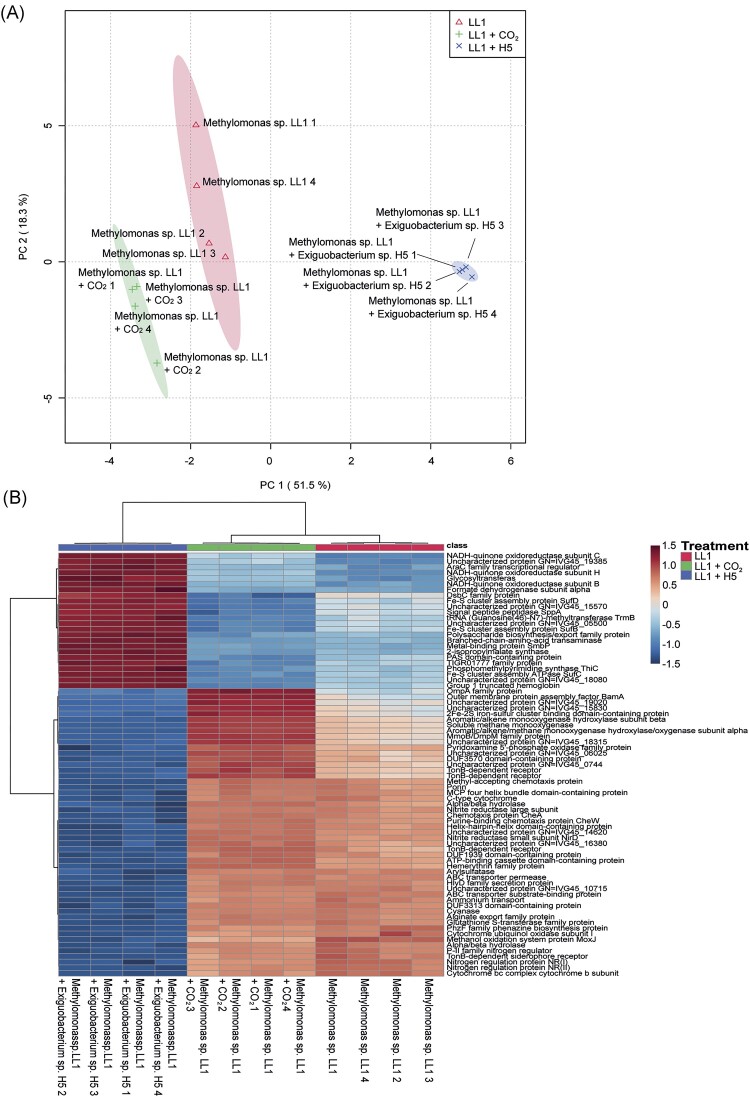
PCA and comparative heatmap visualization of the proteomics approach. (A) PCA plots of the proteomics composition between three different treatments: (i) *Methylomonas* sp. LL1 (red and triangle symbols), (ii) *Methylomonas* sp. LL1 + *Exiguobacterium* H5 (blue and x), and (iii) *Methylomonas* sp. LL1 with the addition of 5% CO_2_ in the headspace (green and cross). (B) Heatmap visualization and clustering analysis of the TOP 75 proteins that significantly differ in their expression between the three treatments shown in the PCA.

Overall, we identified 2605 proteins for *Methylomonas* sp. LL1, 1586 proteins for *M. oxydans* H1, and 1590 proteins for *Exiguobacterium* sp. H5. For *Methylomonas* sp. LL1 we found 115 proteins that were upregulated (log_2_ > 1 and *P*-values < .05) when grown together with *Exiguobacterium*, while 178 proteins were downregulated (Supplementary Table 3). The incubation with elevated CO_2_ led to the detection of 40 upregulated proteins and 46 downregulated proteins. After the growth together with *Methylomonas*, the proteome of *Exiguobacterium* sp. H5 only revealed eight upregulated and seven downregulated proteins. Similarly, the proteome of *Microbacterium* only showed seven upregulated proteins and no downregulated proteins after growth together with *Methylomonas*. Given these results, it was not possible to identify or predict any proteomic interplay between the two heterotrophs and *Methylomonas*. (Supplementary Tables 4 and 5). All three *Methylomonas* sp. LL1 proteomes were clearly separated in the PLS-DA analyses (Fig. 3A), with the proteome of *Methylomonas* sp. LL1 grown under elevated CO_2_ being more similar to the proteome of *Methylomonas* sp. LL1 grown alone than in combination with one of the heterotrophs. Since the incubation of *Methylomonas* sp. LL1 in combination with *M. oxydans* H1 showed no growth, these samples could not be used for a proteomic analysis. The heat maps (Fig. 3B) of the TOP75 proteins significantly differed (*P* < .05) between the treatments. In particular, the proteome of *Methylomonas* sp. LL1 grown in the presence of *Exiguobacterium* sp. H5 was remarkably different from those of *Methylomonas* sp. LL1 grown alone or with CO_2_ amendment. A pathway analysis (GSEA) of the differentially expressed proteins showed that in cultures of *Methylomonas* sp. LL1 grown together with *Exiguobacterium* sp. H5, the CH_4_, fatty acid and pyramidine metabolisms had been downregulated relative to cultures in which *Methylomonas* sp. LL1 was grown alone (Table 2). In *Methylomonas* sp. LL1 cultures supplemented with additional CO_2_, the expression of genes involved in the biosynthesis of valine, leucine and isoleucine, and the 2-Oxocarboxylic acid metabolism were significantly downregulated, while genes involved in CH_4_ and glutathione metabolism, bacterial chemotaxis, degradation of aromatic compounds and two-component systems were upregulated relative to cultures in which *Methylomonas* sp. LL1 was grown alone (Table 2).

**Table 2.** KEGG-based proteomic GSEA shown for the *Methylomonas* sp. LL1 treatments with Exiguobacterium (H1) and 5% CO_2_ in comparison.

Treatment	Pathway^1^	Gene size	NES^2^	NOM^3^ *P*-value	FDR^4^ *q*-value
***Methylomonas* sp. LL1 alone versus with *Exiguobacterium* H1**	Carbon metabolism	94	−2.23	<.001	0.011
	CH_4_ metabolism	52	−2.07	<.001	0.011
	Microbial metabolism in diverse environments	153	−2.07	<.001	0.011
	Fatty acid biosynthesis	13	−1.59	<.001	0.111
	Fatty acid metabolism	13	−1.59	<.001	0.089
	Pyrimidine metabolism	27	−1.5	<.001	0.180
***Methylomonas* sp. LL1 alone versus with + CO_2_**	Valine, leucine, and isoleucine biosynthesis	12	−1.88	<.001	0.054
	2-Oxocarboxylic acid metabolism	24	−1.83	<.001	0.046
	CH_4_ metabolism	52	2.23	<.001	0.018
	Nitrogen metabolism	18	1.75	<.001	0.043
	Bacterial chemotaxis	51	1.67	<.001	0.076
	Carbon metabolism	94	1.63	<.001	0.073
	Microbial metabolism in diverse environments	153	1.62	<.001	0.064
	Glutathione metabolism	12	1.62	.019	0.057
	Degradation of aromatic compounds	8	1.55	<.001	0.078
	Two-component system	106	1.52	<.001	0.080
	Phenazine biosynthesis	3	1.51	<.001	0.078
	ABC transporters	32	1.42	<.001	0.140

1 Upregulated pathways have a positive NES score, while downregulated pathways have a negative score in the first mentioned strain, respectively. Pathways with a *P*-value <.05 and an FDR *q*-value < 0.25 are shown.

2 NES = normalized enrichment score; ^3^NOM = nominal; ^4^FDR = false discovery rate.

### Volatolome analyses

#### Negative effects of the volatolome on the growth of Methylomonas

**Figure 4.**
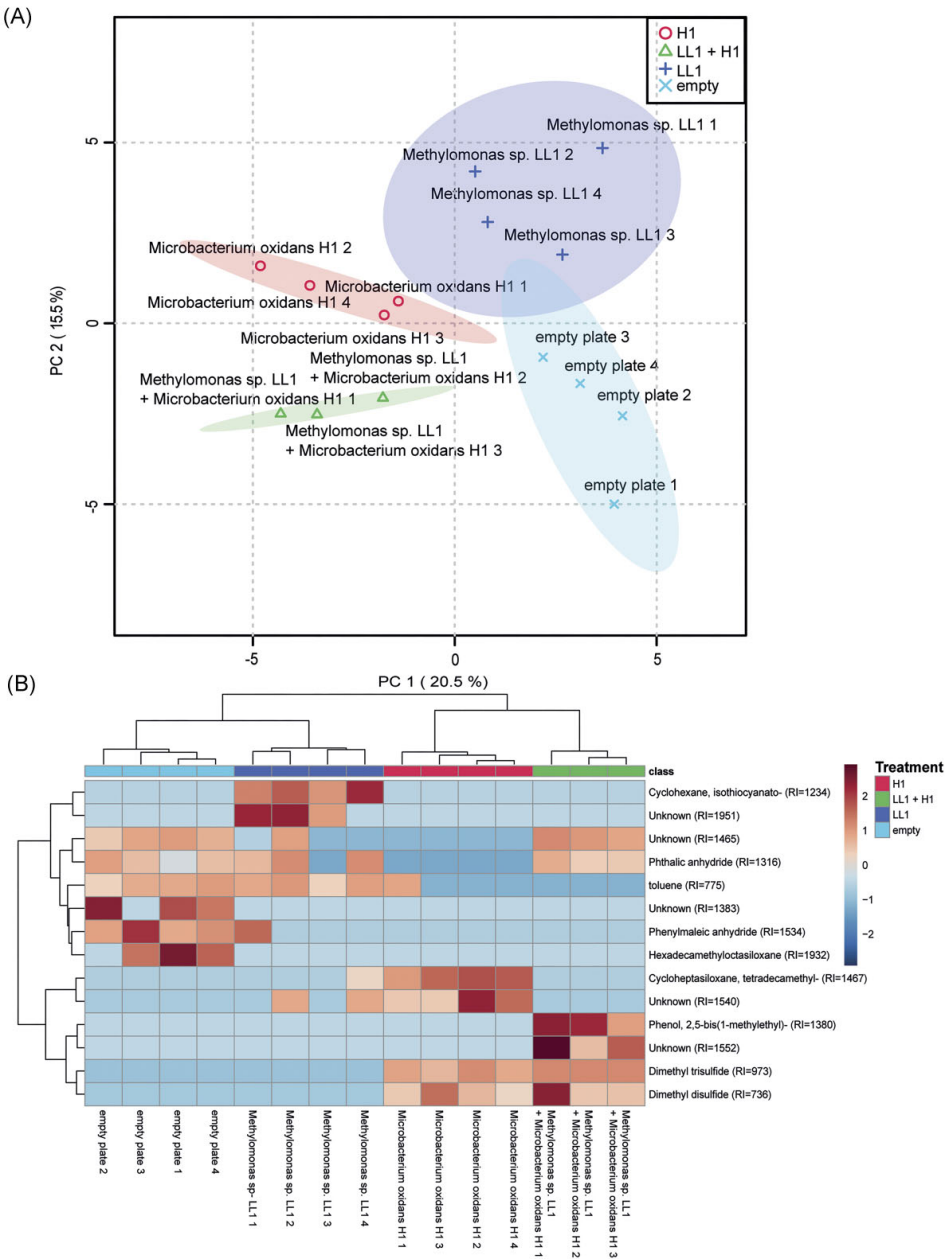
PCA and comparative heatmap visualization of the volatile analysis results for *Methylomonas* sp. LL1 incubated (i) alone (blue and cross) and (ii) in combination with *M. oxydans* H1 (green and triangle), as well as for (iii) *M. oxydans* H1 alone (red and circle) and (iv) an empty Petri dish plate that acted as a negative control (light blue and x). (A) PCA plots of the volatile composition between the different treatments. (B) Heatmap visualization and clustering analysis of all the volatiles that significantly differed between the treatments.

The volatolome differed between all the treatments (Fig. 4A, Supplementary Table 6), with 14 volatiles been identified as significantly different between treatments (Fig. 4B). Dimethyl disulphide and dimethyl trisulphide were only present in samples with *M. oxydans* H1, while cyclohexane was only detected when *Methylomonas* sp. LL1 was grown alone. In addition, a phenol-like compound and an unknown compound were detected on plates where *M. oxydans* H1 and *Methylomonas* sp. LL1 were grown together, while these compounds were not present when *M. oxydans* H1 and *Methylomonas* sp. LL1 were grown alone.

#### Positive effects of the volatolome on the growth of Methylomonas

**Figure 5.**
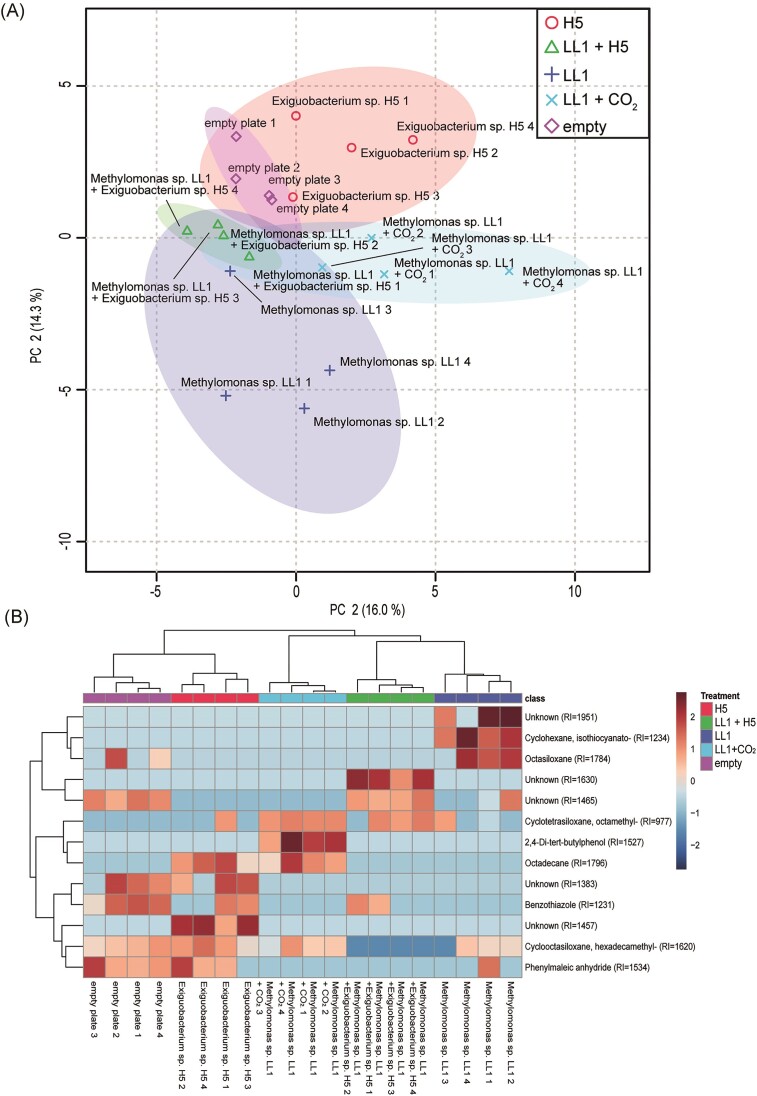
PCA and heatmap analysis of the volatile analyses from *Methylomonas* sp. LL1 alone (blue and cross) and in combination with *Exiguobacterium* sp. H5 (green and triangle) and additional 5% CO_2_ in the headspace (light blue and x) as well as from *Exiguobacterium* H5 alone (red and circle), and an empty Petri dish plate as a negative control (pink and diamond). (A) PCA plots of the volatile composition between the different samples. (B) Heatmap visualization and clustering analysis of all significantly different volatiles between the different samples.

The volatile compounds emitted from plates where the growth of *Methylomonas* was stimulated were not as distinct as those from plates where growth was inhibited (Fig. 5A). Still, we observed 13 significantly different VOCs between the treatments (Fig. 5B). We found an unknown compound that was only present when *Methylomonas* sp. LL1 was growing together with *Exiguobacterium* sp. H5. By contrast, 2,4-di-tert-butylphenol was only detected when the methanotroph was growing in the presence of additional CO_2_.

## Discussion

This study explored the mechanisms and effects of the interaction between methanotrophic and heterotrophic bacteria, which occur naturally together in the environment (Stock et al. 2013, Ho et al. 2014). Our findings suggest that the release of VOCs by heterotrophic bacteria can affect the growth and CH_4_ uptake of methanotrophs positively or negatively depending on the heterotrophic species present.

The coexistence and interactions of these microbial groups, once considered separate entities in ecological models, have emerged as pivotal drivers influencing the fate of CH_4_, a potent GHG, and the flow of carbon in diverse habitats. Methanotrophs, by virtue of their unique metabolic capability to oxidize CH_4_, interface directly with heterotrophic communities reliant on alternative carbon sources by exuding methanol and other metabolites (Stock et al. 2013). The resulting interactions, ranging from cooperative mutualism to competitive antagonism, yield a complex network of metabolic exchanges and dependencies (Ho et al. 2016, Veraart et al. 2018). Unravelling the dynamics and mechanisms of these relationships holds profound implications for ecosystem functioning, carbon cycling, and global climate regulation.

Next to exchange of primary metabolites. Volatile compounds derived from secondary metabolism have been shown to play a role in the interaction of several methanotrophic and heterotrophic species (Veraart et al. 2018, Puri 2019, Kilic 2021). We aimed to underpin our earlier investigations (Veraart et al 2018) with more mechanistic information by exploring this interaction further using a proteomic approach to observe the impact of these VOCs on the metabolic activities of the methanotrophs and heterotrophs. We also wanted to investigate whether the effect of an interaction between heterotrophs and methanotrophs is mainly due to the additional CO_2_ provided by the heterotrophs or is actually triggered by the release of VOCs. We obtained very different results depending on the interacting species, but also a difference between the addition of solely CO_2_, as proxy for a respiring heterotroph, and the presence of a real respiring microbe. While growth of *Methylomonas* sp. LL1 was inhibited in the presence of *Microbacterium’s* volatolome, growth and activity were stimulated when exposed to *E. undae*. Addition of CO_2_ caused a similar stimulation of *Methylomonas* sp. LL1 on growth and CH_4_ uptake, but the accompanying proteomics data of *Methylomonas* sp. LL1 differed significantly between addition of CO_2_ and presence of *Exiguobacterium*, suggesting different underlying mechanisms of the stimulation observed.

### Inhibition of growth of *Methylomonas* by *Microbacterium*

We observed a complete inhibition in growth of *Methylomonas* directly from the beginning of the incubation when exposed to the volatolome of a growing *Microbacterium*, without direct contact. Volatolome analyses led to the conclusion that sulphur compounds [dimethylsulphide (DMS) and dimethyldisulphide (DMDS)] were the agents inhibiting *Methylomonas. Microbacterium* strains have been observed to produce DMDS and DMS, eliciting e.g. plant growth promoting effects, like increased root biomass and a more extensive root system as well as increased shoot biomass (Cordovez et al. 2018, Ballot et al. 2023). It was suggested that the VOCs emitted by *Microbacterium* spp. act as signalling molecules, triggering physiological responses in plants that enhance growth and nutrient uptake. DMS and DMDS are well-known and ubiquitous bacterial volatiles (Ryu et al. 2020) and have also been demonstrated to influence methanotrophic growth and activity, albeit with mixed effects on different methanotrophs (Veraart et al. 2018). While *Methylobacter luteus* showed a stimulation in CH_4_ uptake, but no effect on growth, after growing together with the DMS and DMDS producing heterotroph *Pseudomonas mandelii, Methylocystis parvus* exhibits a contrasting effect with a stimulation in growth, but a decrease in CH_4_ uptake. However, a complete inhibition of growth and activity as in our study was not observed (Veraart et al. 2018). The volatile analyses in this study clearly showed that specifically sulphur compounds like DMS and DMDS were produced by *P. mandelii* and seem to play a role in the interaction. *Methylophaga sulphidovorans* is able to convert DMS to carbon dioxide and thiosulfate. This heterotrophic bacterium, which uses the ribulose monophosphate (RuMP) route for carbon assimilation, can also use sulphide as an additional energy source (Zwart et al. 1996). Furthermore, a large variety of bacteria are capable of oxidizing DMS to DMSO, provided an additional carbon source is present (Zhang et al. 1991). The oxidation can be carried out for example by the ammonium monooxygenases, with its similarity to the pMMO there could be potentially also methanotrophic oxidization of DMS (Fuse et al. 1998). While DMS might be beneficial for certain bacteria, our results show that, at least for *Methylomonas*, the presence of DMS and DMDS has negative effects. In a recent study analysing the methanotrophic community in landfill cover soils, a negative effect of higher DMS concentrations was observed (Wang et al. 2023). The relative abundance of *Methylocaldum* decreased and the CH_4_ oxidation rate was reduced, while at the same time the relative abundance of *Methylobacter* and *Crenothrix* increased. In the same study, a metagenomic analysis of these soils demonstrated a reduction in the *mmo* and *mxa* genes involved in CH_4_ uptake and CH_4_ assimilation. Since the growth of *Methylomonas* was completely inhibited in our study, we could not perform a proteomic analysis to determine possible inhibition of specific metabolic pathways such as sulphur oxidation or reduction. To understand how DMS, DMDS or other sulphur compounds can interact with different bacteria strains needs to be further analysed to understand their impact in the environment.

However, important to keep in mind is that in the natural context, there are multiple microbes around producing and possibly consuming volatiles which makes that our results of one on one interaction have to be put in context. With respect to this, when looking at relevant scales to microbes, e.g soil pore or aggregate scale, than the numbers of cells really interacting with each other can be very small and even one to one (Amelung et al. 2024), strengthening the environmental realism of our results. On the other hand, volatiles in soil can travel over cm's distance and can even obvious one to one interaction may be influenced from elsewhere (Schulz-Bohm et al. 2018).

### Stimulation of activity of *Methylomonas* by *Exiguobacterium* and increased CO_2_ concentrations

The results of our study clearly showed an increase in growth and CH_4_ uptake rates by *Methylomonas* sp. LL1 growing together with *Exiguobacterium undea* or by addition of CO_2_ solely. To investigate the mechanisms behind these stimulatory effects we conducted a proteomics analysis. In a recent study, growth of *Methylocystis parvus* was also promoted by additional CO_2_ and the presence of heterotrophs, but not CH_4_ uptake rates (Veraart et al. 2018). In the same study *Methylobacter luteus* showed the complete opposite effect, with an increase in CH_4_ uptake rates, but a constant growth compared to the control. In both, Veraart et al. (2018) and the current study the effects of the presence of a heterotroph or the addition of CO_2_ seem to be quite similar. However, the proteomics data in this study differ significantly when *Methylomonas* is grown alone, with the addition of extra CO_2_ or together with *Exiguobacterium*. The latter results in a significantly different proteome of *Methylomonas* than the first two treatments, leading to the assumption that the stimulation by the volatolome of a heterotroph is not solely caused by the presence of CO_2_, but probably by other VOCs.

Stimulation of growth by CO_2_ maybe caused by fixation using the reductive glycine cycle, a pathway which in theory can also be used by some methanotrophs (Tveit et al. 2019, Claassens 2021, Nguyen et al. 2021). However, in our proteomics analyses the enzymes involved in the reductive glycine cycle did not show differences between the different samples, but all involved enzymes were detected in all samples (Supplementary Table 3). Nevertheless, there were two very clear differences in the proteome analyses in the CH_4_ processing pathways. While the sMMO of *Methylomonas* is upregulated after the addition of CO_2_, it is downregulated growing together with *Exiguobacterium*. An opposite trend is observed for the RuMP pathway, which is upregulated alongside *Exiguobacterium* and downregulated with additional CO_2_. This suggests a regulation of pMMO versus sMMO expression, possibly in response to higher CO_2_ concentrations and changing redox conditions, similar to the regulation seen with copper. The downregulation of RuMP in the presence of *Exiguobacterium* might indicate a shift to directing more CH_4_ carbon into the electron transport chain for additional energy generation, especially when essential compounds are provided by the heterotroph (Holmes et al. 2018). Additionally, there is also a possibility that methanotrophs (*Verrucomicrobia*) can grow as autotrophs together with H_2_ as a sole electron source (Mohammadi et al. 2019). It was shown that the NiFe-hydrogenase that is needed for this conversion is also present in our *Methylomonas* strain (de Assis Costa et al. 2021). However, we did not detect an upregulation of the NiFe-hydrogenase in our proteomics data, even though it was detectable. There are *Methylomona*s strains which are capable of fixing CO_2_ through the PEP carboxylase, pyruvate carboxylase, and acetyl-CoA carboxylase (Nguyen et al. 2018). However, it seems that our *Methylomonas* strain only harbours the first two carboxylases but not the latter one.

Remarkably, there are multiple studies in rice paddies and grasslands showing that elevated CO_2_ (eCO_2_) concentrations have a positive impact on growth of methanotrophs and the rate of uptake of CH_4_ (Yu et al. 2018, Qian et al. 2020, 2022). In these studies type I methanotrophs were the benefactors of this eCO_2_ compared to type II (Liu et al. 2022). The authors of these studies hypothesized that there is an indirect effect of CO_2_ driving an increase in DOC and root growth, providing a more friendly environment for methanotrophs by an increase in available CH_4_ and increase of oxic zones. However, our data point to a direct effect driving the observed results, of which the principal mechanisms of the direct effect of CO_2_ on the sMMO expression in *Methylomonas*, we cannot explain. Speculative, the effect of CO_2_ could be through reducing equivalents that the sMMO needs to initiate the first step of CH_4_ metabolism. To date the exact source of reducing equivalents for sMMO is still not fully understood, but it is thought to involve flavoproteins and iron–sulphur proteins that participate in electron transfer reactions. It utilizes a cofactor called a dinuclear iron center, often referred to as the diiron center, which plays a crucial role in the catalytic reaction (Whittington and Lippard 2001). The reducing equivalents generated through cellular metabolism are utilized by MMO to activate molecular O_2_ and initiate the oxidation of CH_4_. There are some studies that show that formate dehydrogenase (FDH) enzymes, cytochrome c, or succinate dehydrogenase flavoprotein subunit enzymes are important in the uptake and reduction of CO_2_ in the cell (Wan et al. 2016, Ruiz-Valencia et al. 2020, Alothaim 2023). Even though we detect these enzymes in the proteome of *Methylomonas*, they do not show a specific regulation in the incubation with CO_2_. The handling of the samples can influence the proteomics analyses. The setup of these experiments may introduce bias in the analyses due to the examination of cells in various growth stages within a single colony. So maybe it is a matter of timing, since for example for the FDHs it was shown that this could be used as a precursor for methanol production from CH_4_ by methanotrophic bacteria (Ruiz-Valencia et al. 2020), which speeds up the whole first step in CH_4_ oxidation, we may have missed this with our sampling approach. Besides the indications from other bacterial species there are no studies for methanotrophs about transport or uptake of CO_2_, therefore more studies are needed that specifically focus on this interaction, especially since under natural conditions, CO_2_ can reach up to 5% in in soils and especially in rhizosphere environments (Button et al. 2023, Bereswill et al. 2024).

The impact of *Exiguobacterium* on the function and growth of methanotrophs appeared to exhibit similarities to the effects of CO_2_, as previously mentioned. While both stimulate the growth, this effect was more than doubled with the added CO_2_, with the same CH_4_ uptake rates. This also hint into the direction that the CO_2_ was directly used as a carbon source. The amount of CO_2_ respired by *Exiguobacterium* is probably nowhere near the amount of CO_2_ added directly. Unfortunately, the CO_2_ production and consumption was not measured during the course of the experiment, therefor, the sudden exposure to high and gradually increasing CO_2_ is different, which may lead to different responses of which we do not known what consequences it may have on methanotrophic growth and activity. However, our analysis of volatolomics and proteomics data revealed also divergent indications from those associated with CO_2_. While we successfully detected specific VOCs in the volatolome analysis, these compounds were found exclusively in the interaction between *Methylomonas* and *Exiguobacterium*, yet their precise identification could not be determined. However, the proteomics analysis provided insights into the upregulation observed in this interaction, revealing that it was not the sMMO like the effect with CO_2_, but rather the RuMP pathway that exhibited upregulation. The RuMP pathway represents one of the three primary pathways employed by methanotrophic bacteria to assimilate CH_4_ (Hanson and Hanson 1996, Chistoserdova and Lidstrom 2013, Chistoserdova and Kalyuzhnaya 2018). In this pathway, CH_4_ is oxidized to formaldehyde, which is subsequently converted to ribulose-5-phosphate through a series of enzymatic reactions. Ribulose-5-phosphate can then enter the pentose phosphate pathway, providing the cell with energy and biosynthetic intermediates. The RuMP pathway is widely utilized by methanotrophs and plays a critical role in the global carbon cycle by converting CH_4_ into biomass. However, the mechanisms causal of the stimulation of the RuMP pathway through the interaction with *Exiguobacterium* remain uncertain. Additionally, we observed an upregulation of the pyrroloquinoline quinone (PQQ)-dependent methanol dehydrogenase and an increase in PQQ production. This suggests that the pathway from methanol to formaldehyde, and subsequently to the RuMP pathway, may be influenced by the growth of both heterotrophic bacteria and methanotrophs. It is possible that the heterotrophs provide reducing equivalents at an accelerated rate, enabling the methanotrophs to gain an advantage in the initial uptake and oxidation of CH_4_, consequently leading to higher metabolic activity downstream. However, further investigations are needed to elucidate the precise mechanisms involved, as the optimal sampling time or missing connecting information may have influenced the measurements.

### Outlook

The current study explored the interaction between methanotrophic and heterotrophic bacteria, as well as high concentrations of CO_2_, and their effects on the functionality of the methanotrophs (growth, CH_4_ oxidation, and carbon cycling). Understanding the dynamics of the interaction between methanotrophic and heterotrophic bacteria can have implications for ecosystem functioning, carbon cycling, and global climate regulation. Methanotrophs play a crucial role in mitigating the effects of CH_4_, a potent GHG, by oxidizing it. The study highlights that the interaction between these bacteria can influence the fate of CH_4_ and the flow of carbon in diverse habitats. The findings suggest that the release of VOCs by heterotrophic bacteria can affect the growth and CH_4_ uptake of methanotrophs. Different VOCs, such as dimethyl-sulphide and dimethyl-disulphide, were found to have varying effects on different methanotroph species. Some VOCs stimulated growth and CH_4_ uptake, while others inhibited them. These findings indicate that the presence and composition of VOCs in the environment can impact the activity of methanotrophs and, consequently, the rate of CH_4_ oxidation. Moreover, the study highlights the role of CO_2_ in the interaction between bacteria. Elevated CO_2_ concentrations were found to have a positive impact on the growth of methanotrophs and their CH_4_ uptake rates. This suggests that increased CO_2_ levels, such as those associated with climate change, can potentially enhance the activity of methanotrophs and contribute to the reduction of CH_4_ emissions. Overall, the study emphasizes the importance of understanding the interactions between methanotrophic and heterotrophic bacteria in order to comprehend their influence on CH_4_ oxidation, carbon cycling, and ultimately, global climate regulation. These insights can inform strategies for mitigating GHG emissions and managing carbon in the environment.

## Supplementary Material

fiae112_Supplemental_File
